# Brexpiprazole augmentation in patients with a resistant obsessive compulsive disorder with psychotic features: an observational study in a psychiatric rehabilitation center

**DOI:** 10.1192/j.eurpsy.2025.1711

**Published:** 2025-08-26

**Authors:** F. Franza, A. Franza, F. Pellegrino, G. Del Buono, A. Ricca

**Affiliations:** 1 Psychiatric Rehabilitation Center “Villa dei Pini”, Avellino; 2 Neamente Neuroscience Study Center, Avellino - Naples, Italy

## Abstract

**Introduction:**

The relationship between obsessive-compulsive disorder and psychotic disorders has been frequently observed. Subject to diagnostic and therapeutic controversies, this association is a challenge for clinicians. Several scientific studies have observed a frequency of approximately 12% of this association in patients affected by schizophrenia. The efficacy of augmentation of SGAs with antidepressants in treatment-resistant OCD is demonstrated. Several studies have shown the efficacy of some dopamine receptor partial agonists (DRPAs) (aripiprazole and cariprazine) in augmentation with SSRIs in zOCD-psychotic features treatment-resistant. Brexpiprazole is a DRPAs with serotoninergic 5-HT1A agonism and 5-HT2A receptor antagonist.

**Objectives:**

To evaluate the efficacy of brexpiprazole in augmentation with SSRIs in OCD patients with psychotic features resistant to pharmacological treatment.

**Methods:**

Eleven patients (6 females, 5 males; mean total age (42.818 yrs ±12.679)) were recruited into our observational study. Affected by Obsessive Compulsive Disorder with psychotic features (based on the DSM-5-TR criteria). All patients were assessed using the SCID-5-CV. Levels of insight were assigned using DSM-5-TR specifiers. A detailed interview was conducted containing information on the demographic profile of the patients. All patients were administered the following rating scales: BPRS, Y-BOCS, GAF, CGI-S, at baseline (T0), after one month (T1), two months (T2), six months (T3), and one year (T4). All patients were administered bexpiprazole, replacing other SGAs associated with SSRIs (see Table 1).

**Results:**

Table 2 and the graphic show the results obtained with our study. A statistically significant reduction of the mean total score was observed in the BPRS, Y-BOCS scales, an increase in the mean total score with the GAF. An improvement in the mean total score was also observed with the CIG-S. In all the scales used, the ANOVA results indicate that at least two of the repeated measures differed significantly. The preliminary data also indicate adequate safety and effectiveness.

**Image 1:**

**Figure:**
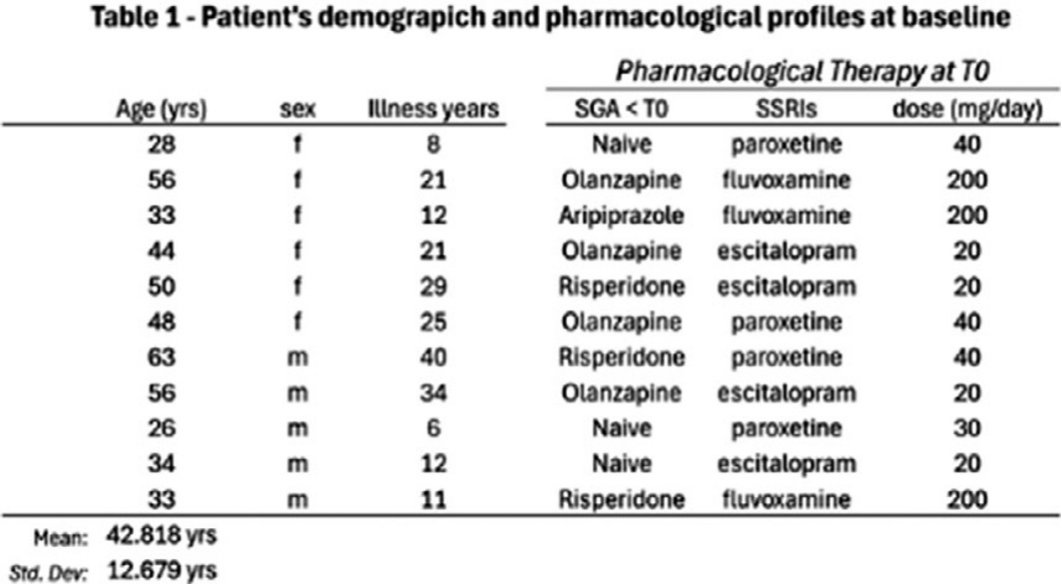

**Image 2:**

**Figure:**
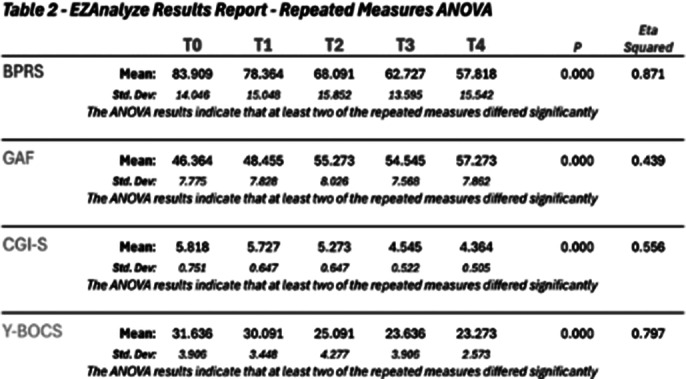

**Image 3:**

**Figure:**
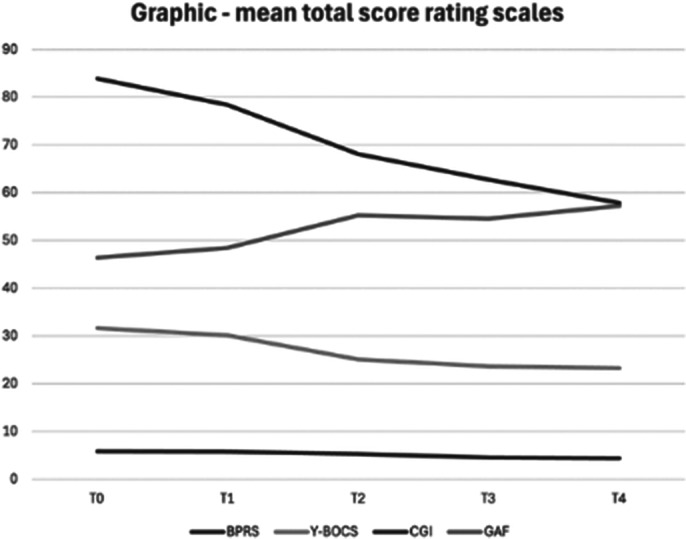

**Conclusions:**

Our small observational study aimed to evaluate the efficacy of brexpiprazole in a group of patients affected by OCD with psychotic features. Despite the small sample analyzed, the results of our study point towards a possible use of brexpiprazole in this group of patients affected by OCD with psychotic features during normal routine clinical practice.

**Disclosure of Interest:**

None Declared

